# Multidrug-resistant CTX-M-15-positive *Klebsiella pneumoniae* ST 307 causing bacteremia via gut translocation in a dog

**DOI:** 10.3389/fvets.2023.1275822

**Published:** 2023-10-26

**Authors:** Ji-Yeon Hyeon, Yun-Jeong Choi, Min-Jung Jung, Dong-Hun Lee, Chang-Seon Song, Jung-Hyun Kim

**Affiliations:** ^1^Department of Veterinary Medicine, College of Veterinary Medicine, Konkuk University, Seoul, Republic of Korea; ^2^Department of Veterinary Internal Medicine, College of Veterinary Medicine, Konkuk University, Seoul, Republic of Korea; ^3^KHAV Co., Ltd., Seoul, Republic of Korea

**Keywords:** *Klebsiella pneumoniae*, ST 307, bacteremia, multidrug-resistance, whole genome sequencing

## Introduction

*Klebsiella pneumoniae* (*K. pneumoniae*) has been commonly associated with human nosocomial infections and has recently gained special attention as a clinically important pathogen in companion animals (1). In companion animals, *K. pneumoniae* causes extraintestinal infections, such as urinary tract infections, pyometra, upper respiratory tract infections, and bloodstream infection (septicemia) (2–4). In recent years, multidrug-resistant and hypervirulent *K. pneumoniae* have spread widely as a critical public health threat in the world (4). Of these, *K. pneumoniae* sequence type 307 (ST 307) has emerged as a new multidrug-resistant *K. pneumonia* clone worldwide in both humans and animals (5). There are several KP ST307 outbreaks in humans globally; the Netherlands in 2016 (6), Germany in 2019 (7), and South Korea in 2015 (8) and 2018 (9). *K. pneumoniae* ST 307 infections have been reported in dogs and cats suffering from urinary tract infections (1, 2, 10). In South Korea, *K. pneumoniae* ST 307 is one of two main clones of *K. pneumoniae* isolates from companion animals in Lee et al.'s study (11). Recent studies reported multidrug-resistant *K. pneumoniae* ST 307 infections in companion animals, but there is limited study on their genetic characteristics such as virulence profiles and phylogenetic relationship using whole genome sequence (WGS) (1, 4). In addition, genetic characteristics of *K. pneumoniae* strains that cause bacteremia by gastrointestinal system have rarely been investigated in dogs. Inter-species transmission of antimicrobial resistant bacteria between people and household pets, such as dogs and cats, is an emerging global public health problem. Such cross-transmission events have garnered concern in light of their implications for public health and underscore the urgency of genomic analysis as an essential tool in understanding and identifying of this potential threat (1).

Bacteremia has been defined as the presence of viable bacteria in the bloodstream. Genitourinary and gastrointestinal systems, pneumonia, pyometra, and wounds are common sources of bloodstream infection (12, 13). Several mechanisms that promote the translocation of indigenous bacteria from the gastrointestinal systems have been identified, such as intestinal bacterial overgrowth, deficiencies in host immune defenses, and intestinal mucosal barrier damage (14). For example, a severe outbreak of *K. pneumonia* enteritis in a kennel of Bordeaux mastiffs, resulting in septicemia and death, has been reported in a previous study (15). The study assumed that the systemic *Klebsiella* infection most likely originated from the gastrointestinal infection based on the gastrointestinal symptoms, the number of dogs affected, the dietary history, and the necropsy findings (15). However, molecular epidemiological analysis of the *K. pneumoniae* isolates had not been performed in this study.

Whole-genome sequencing technique yields insight into strain relatedness, by assessing distances from one another in single nucleotide polymorphisms (SNPs) and has been used in epidemiological investigations (16). By comparing the genetic similarity between the bacteria in the bloodstream and the bacteria isolated from another site, researchers can identify the source of bacteremia using WGS. In this study, we report the two *K. pneumoniae* ST 307 isolates from blood and fecal samples of a dog with bacteremia and enteritis in South Korea. We analyzed the presence of antibiotic resistance genes and virulence gene profiles of the isolates, and genetic relationship between the isolates to identify the source of the bloodstream infection in the dog. In addition, we compared the virulence profile and phylogenetic relationship with other *K. pneumoniae* ST 307 from dogs and cats.

## Materials and methods

### Bacterial isolation, identification, and antibiotic susceptibility test

A 12-year-old spayed female poodle dog weighing 3.5 kg was referred to the Veterinary Teaching Hospital at Konkuk University (Seoul, South Korea) for evaluation of a 1-month history of diarrhea, fever, lethargy, and anorexia in September 2022. Blood, urine, and fecal samples collected from the dog were submitted to NosVet Laboratory (Gyeonggi-do, South Korea) to isolate the causative agent and antibiotic resistance test. Two *K. pneumoniae* isolates were isolated from the blood (KP-B) and fecal (KP-F) samples, and the urine samples were negative for bacterial culture. The isolates were identified using MALDI-TOF, and antibiotic susceptibility of the isolates was determined by the Kirby-Bauer Test disc-diffusion method as recommended by the Clinical and Laboratory Standards Institute for a consensus interpretive criterion (17).

### Whole genome sequencing

For WGS, genomic DNA was extracted from pure cultures of the KP-B and KP-F using the MagNA Pure 96 DNA and Viral NA Small Volume Kit on the MagNA Pure 96 instrument (Roche Applied Sciences, Germany) according to the manufacturer's instructions. Sample DNA concentrations were determined using a Qubit BR dsDNA assay kit (Invitrogen, Carlsbad, CA), and DNA (0.2 ng/μl)were used for the library preparation using the Illumina Nextera XT DNA Library Prep Kit (Illumina, San Diego, CA) as previously described (18). The library pool (500 μl of the 10 pM libraries) was loaded into the MiniSeq High Output Reagent cartridge (300 cycles) (Illumina). The paired FASTQ files were base-called from the Illumina raw sequence read data.

### Whole genome sequence analysis

The raw reads were adapter-trimmed for known Illumina adapters and quality-trimmed with Bbduk (https://sourceforge.net/projects/bbmap) (Q > 20 and minimum length >50), and trimmed reads were *de novo* assembled using the SPAdes 3.15.5 (19) with its default settings in Geneious Prime 10 Software (https://www.geneious.com/). The assembled contigs with coverage of <5 × and size below 300 bases were removed. To confirm species identification, the 16S rRNA regions in the assembled contigs of the isolates were predicted by barrnap (Galaxy Version 1.2.1), and the nearest-neighbor species with >99% identity were first searched using the BLASTn on the NCBI database (https://blast.ncbi.nlm.nih.gov/Blast.cgi) with the default parameters for each 16S rRNA sequence. MLST 2.0 (Multi-Locus sequence typing) was used to determine the sequence type of the isolates. The presence of acquired antimicrobial resistance genes and chromosomal mutations in the *gyrA, gyrB, parC*, and *parE* genes were determined using ResFinder 4.1 (https://cge.food.dtu.dk/services/ResFinder-4.1/) with settings of a threshold of 90%, and a minimum length of 60% with the assembled contigs.

For comparative genomic analysis, all available genome sequences of *K. pneumoniae* ST 307 from cats and dogs (*n* = 37) were downloaded from BV-BRC (https://www.bv-brc.org/) and BIGSdb-Pasteur (https://bigsdb.pasteur.fr/). WGS of two *K. pneumoniae* ST 307 isolates (KP 44 and KP 45) from dogs referred to the Veterinary Medical Teaching Hospital of Konkuk University were also included for the subsequent WGS analysis. Sample information of the genomes is listed in Supplementary Table S1. The virulence profiles of the *K. pneumoniae* ST 307 isolates including our isolates were compared after annotation using the BV-BRC annotation server. The sequences were annotated using the BV-BRC annotation server (https://www.bv-brc.org/) with default parameters. Protein annotations involved in virulence factors of the annotated genomes were downloaded using the specialty genes service of BV-BRC with the Virulence Factor Database (VFDB) filter, and the genes with their classification were used for subsequent analyses. For phylogenetic analysis, a total of 41 *K. pneumoniae* ST 307 including our isolates were used for SNP analysis. A completed genome of *K. pneumoniae* ST 307 strain Z0117KP0004 from a dog from South Korea (accession no. GCA_023657855.1) was used as a reference genome. High-quality SNPs analysis and maximum likelihood (ML) phylogenetic tree construction were conducted using the default quality filters in CSI phylogeny (20).

### Whole genome sequence analysis results

*K. pneumoniae* ST 307 isolates were isolated from blood (KP-B) and feces (KP-F) from the dog with bacteremia and enteritis in this study. The KP-B and KP-F isolates revealed the identical phenotypic and genotypic antibiotic resistance (Table 1). They were resistant to all antibiotics tested except aminoglycosides (gentamicin, amikacin), amoxicillin/clavulanic acid, and carbapenems (imipenem) (Table 1). The isolates harbor multiple acquired antibiotic resistance genes; fluoroquinolone and aminoglycoside resistance genes [*aac (6*′*)-Ib-cr*], aminoglycoside resistance genes [*aph (3*′'*)-Ib, aph (6)-Id*], beta-lactam resistance genes (*bla*_CTX − M−15_, *bla*_OXA − 1_, *bla*_SHV − 106_, and *bla*_TEM − 1B_), phenicol resistance gene (*catB3*), trimethoprim resistance gene (*dfrA14*), fosfomycin resistance gene (*fosA*), disinfectant resistance genes (*OqxA, OqxB*), quinolone resistance gene (*qnrB1*), sulphonamide resistance gene (*sul2*), and tetracycline resistance [*tet (A)*] (Table 1). In addition, chromosomal mutations were observed in *acr*R, *omp*K37, *omp*K36, *par*C, and *gyr*A genes (Table 1). The *K. pneumoniae* ST307 has been known as an important human pathogen harboring transferable resistance-conferring genes against carbapenems and newer-generation cephalosporins such as *bla*_KPC − 2_, *bla*_KPC − 3_, *bla*_NDM − 1_, *bla*_OXA − 48_, and *bla*_CTX − M−15_ (1, 5, 7, 9, 21). In previous studies (1, 2, 22, 23), *bla*_CTX − M−15_-carrying *K. pneumoniae* ST 307 was reported as a predominant clone in dogs suggesting the spread of this clone in the animal population. In South Korea, *bla*_CTX − M−15_-positive *K. pneumoniae* ST 307 is prevalent in human isolates from hospitals and has been detected in cases of bacteremia (24). Additionally, it was responsible for one of the two documented *K. pneumoniae* ST 307 outbreaks in humans (9).

**Table 1:** Phenotypic and genotypic antibiotic resistance of the *K. pneumoniae* ST 307 isolates, KP-B and KP-F from a dog with bacteremia in this study.

**Antibiotic class**	**KP-B**		**KP-F**	
	**Phenotype** ^*^	**Genotype**	**Phenotype** ^*^	**Genotype**
**Aminoglycoside resistance**				
Amikacin	S	*aph(3′')-Ib, aph(6)-Id, aac(6′)-Ib-cr*	S	*aph(3′')-Ib, aph(6)-Id, aac(6′)-Ib-cr*
Erythromycin	R		R	
Gentamicin	S		S	
**Beta-lactam resistance**				
Ampicillin	R	*blaCTX-M-15, blaOXA-1, blaSHV-106, blaTEM-1B*	R	*blaCTX-M-15, blaOXA-1, blaSHV-106, blaTEM-1B*
Amoxycillin/Clavulanic acid	I		I	
**Carbapenems**				
Imipenem	S	ompK37 p.I70M, ompK37 p.I128M, ompK37 p.N230G	S	ompK37 p.I70M, ompK37 p.I128M, ompK37 p.N230G
**Cephalosporin resistance**				
Cephalexin	R	ompK36 p.N49S, ompK36 p.L59V, ompK36 p.T184P	R	ompK36 p.N49S, ompK36 p.L59V, ompK36 p.T184P
Cephazolin	R		R	
Cefaclor	R		R	
Ceftazidime	R		R	
Cefotaxime	R		R	
Cefixime	R		R	
Cefpodoxime	R		R	
Cefovecin	R		R	
**Fluoroquinolone**				
Enrofloxacin	R	qnrB1 acrR p.P161R, acrR p.G164A, acrR p.F172S, acrR p.R173G, acrR p.L195V, acrR p.F197I, parC p.S80I, gyrA p.S83F	R	qnrB1 acrR p.P161R, acrR p.G164A, acrR p.F172S, acrR p.R173G, acrR p.L195V, acrR p.F197I, parC p.S80I, gyrA p.S83F
Marbofloxacin	R		R	
**Tetracyclines**				
Tetracycline	R	tet(A)	R	tet(A)
Doxycycline	R		R	
**Sulfonamides**				
Sulfamethoxazole/Trimethoprim	R	sul2	R	sul2
**Macrolide resistance**				
Azithromycin	R		R	
Ofloxacin	R		R	
**Lincosamides**				
Clindamycin	R		R	
**Nitrofuranes**				
Nitrofurantoin	R		R	
**Monobactams**				
Aztreonam	R		R	
**Disinfectant**	ND	OqxA, OqxB	ND	OqxA, OqxB
**Phenicol**	ND	catB3	ND	catB3
**Fosfomycin**	ND	fosA	ND	fosA

^*^R, resistant; S, sensitive; I, intermediate; ND, not determined.

**Figure 1:**
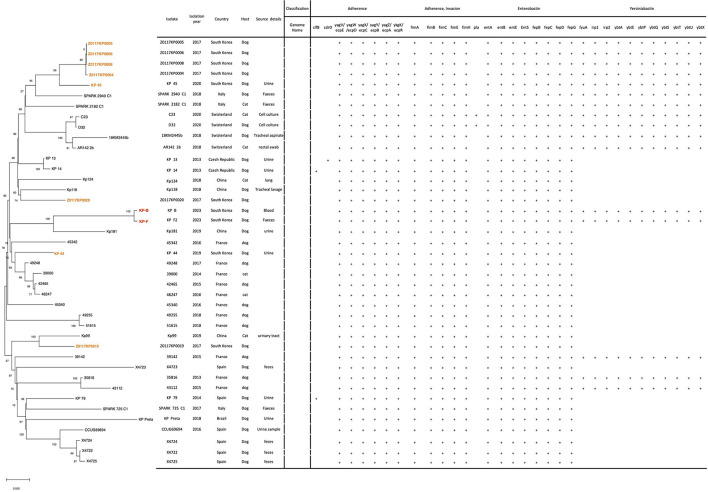
Phylogenetic analysis and virulence gene profiles of *K. pneumoniae* ST 307 isolates from dogs and cats (*n* = 41). Two isolates, KP-B and KP-F, from this study were highlighted in red and eight isolates from South Korea in orange. The phylogeny was rooted at the midpoint. The numerical values on certain tree branches represent 1,000 bootstrap replicate values expressed as a percentage.

A total of 351 sequences of *K. pneumoniae* ST 307 were available in BIGSdb-Pasteur from animals (*n* = 29), environment (*n* = 6), human (*n* = 299), and unknown source (*n* = 17). Additionally, 1,283 sequences were available in BV-BRC from animals (*n* = 31), environment *n* = 3), human (*n* = 1,111), and unknown (*n* = 138). For phylogenetic analysis, 37 WGS of *K. pneumoniae* ST 307 from cats and dogs were downloaded from these databases. The phylogenetic tree of 41 genome sequences of the *K. pneumoniae* ST 307 is shown in Figure 1. The phylogenetic tree revealed that the sequences of the KP-B isolate showed high similarity (99.6%, 4 SNPs, data not shown) with the KP-F isolate. According to the previous study on outbreaks of carbapenem-resistant *Klebsiella* spp. (16), the SNP cut-off values defining isolate relatedness ranged between 0 and 131 SNPs, and intra-patient diversity in isolates ranged between zero and seven SNPs. This result suggests that *K. pneumoniae* in this study translocated into the bloodstream through the gastrointestinal tract, leading to bacteremia. To the best of our knowledge, this is the first report on the utilization of WGS analysis to determine the source of bloodstream infection.

The phylogenetic tree of the of 41 genome sequences of the *K. pneumoniae* ST 307 revealed two lineages, and each lineage comprised the genomes from the Asia, Europe, and South America (Figure 1). The KP-B and KP-F isolates grouped with Kp181 isolated from urine from a dog in China (91.7% of similarity, data not shown) (Figure 1). Five of ten isolates from South Korea, Z0117KP020, KP-B (KP-F), KP 44, and Z0117KP0019, were determined to be singletons in phylogeny, with no observed cluster relationships with other isolates from South Korea. It indicated that *K. pneumoniae* ST 307 has been transferred between countries and become globally disseminated. This has been reported in the comparative analysis of 95 *K. pneumoniae* ST 307 genomes from various sources by Wyres et al. (5).

We analyzed the virulence profile of our *K. pneumoniae* ST 307 isolates caused bacteremia in a dog to examine whether the isolates carry the virulence genes correlated to hypervirulent *K. pneumoniae*; *rmpA/rmpA*2 (regulator of the mucoid phenotype gene A), *magA* (microviscosity-associated gene A), and genes encoding siderophores, such as aerobactin, enterobactin, and yersiniabactin (25–27). Siderophores are small molecules with various affinities for iron, with aerobactin having the lowest affinity and enterobactin having the highest (26). Several studies used whole genome sequencing to investigate the genetic characteristics of the hypervirulent *K. pneumoniae* isolates causing bloodstream infection in humans (25, 26, 28). None of our isolates carried *rmpA/rmpA2* and *magA*, but they encoded genes for siderophores, enterobactin, and yersiniabactin (Figure 1). Our isolates showed the identical virulence profile carrying the genes associate with adherence (*yag*V-*yag*Z, *ykg*K, *fim*A-*fim*C, *fim*E, and *fim*H), enterobactin (*ent*A, *ent*B, *ent*E, *ent*S, *fep*B-*fep*D, and *fep*G), and yersiniabactin (*fyu*A, *irp*1, *irp*2, *ybt*A, *ybt*E, *ybt*P, *ybt*Q, *ybt*S, *ybt*T, *ybt*U, and *ybt*X). All the other *K. pneumoniae* ST 307 isolates also harbored the virulence genes associated with adherence (*yag*V-*yag*Z, *ykg*K, *fim*A-*fim*C, *fim*E, and *fim*H) and enterobactin (*ent*A, *ent*B, *ent*E, *ent*S, *fep*B-*fep*D, and *fep*G), but 14 isolates of them carried the genes encoding yersiniabactin (Figure 1). Therefore, it suggests that the bacteremia in this study might be influenced more by host immune status and the antimicrobial treatment than by the genetic characteristics of the infecting pathogen.

In this study, we report the WGS of *K. pneumoniae* ST 307 causing bacteremia via gut translocation in a dog in South Korea. Intestinal bacterial translocation of the bacteria to the bloodstream was confirmed by SNP analysis of the isolates from blood and fecal samples (4 SNPs) using WGS. The isolates showed multidrug-resistance and harbored multiple antimicrobial resistance genes including *bla*_CTX − M−15_. The virulence gene profiles suggested that the *K. pneumoniae* ST 307 isolates were not hypervirulent *K. pneumoniae* but carried the genes encoding siderophores. This study is the first report on *K. pneumoniae* ST 307 from the bacteremia in a dog and the utilization of the WGS analysis to define the source of the bloodstream infection. It provides valuable reference data for genomic surveillance of new emerging *K. pneumoniae* ST 307 in companion animals alongside other well-known clones. Considering the emergence and rapid dissemination of high-risk multidrug-resistant *K. pneumoniae* in both companion animals and humans, surveillance strategies and genomic studies are essential in human and veterinary medicine.

## Data availability statement

The datasets presented in this study can be found in online repositories. The names of the repository/repositories and accession number(s) can be found below: https://www.ncbi.nlm.nih.gov/, PRJNA956693.

## Ethics statement

Sample collection for bacterial isolation involves procedures or treatments that fall under standard veterinary practices for diagnosing and treating animals, therefore, ethical approval was considered unnecessary. Written informed consent was obtained from the owners for the participation of their animals in this study.

## Author contributions

J-YH: Writing—original draft. Y-JC: Methodology, Writing—original draft. M-JJ: Methodology, Writing—original draft. D-HL: Supervision, Writing—review and editing. C-SS: Supervision, Writing—review and editing. J-HK: Conceptualization, Supervision, Writing—review and editing.

## Supplementary material

Supplementary Table 1 *K. pneumoniae* ST 307 isolates information.
